# A qualitative study of medical-surgical intensive care unit nurses’ experiences in caring for critical patients

**DOI:** 10.1590/1980-220X-REEUSP-2022-0220en

**Published:** 2022-11-14

**Authors:** Burcu Totur Dikmen, Nurhan Bayraktar, Ümran Dal Yılmaz

**Affiliations:** 1 Near East University, Faculty of Nursing, Department of Surgical Nursing, Nicosia, Cyprus.; 2 Atılım University, Health Sciences Faculty, Department of Nursing, Ankara, Turkey.

**Keywords:** Nursing, Nursing Care, Critical Care, Intensive Care Units, Enfermería, Atención de Enfermería, Cuidados Críticos, Unidades de Cuidados Intensivos, Enfermagem, Cuidados de Enfermagem, Cuidados Críticos, Unidades de Terapia Intensiva

## Abstract

**Objective:**

To describe the meaning attributed to nurses’ clinical experience in a medical-surgical intensive care unit in Northern Cyprus.

**Method:**

The qualitative study was conducted in two medical-surgical intensive care units at a university hospital. Data were collected through in-depth interviews with 17 nurses. Giorgi’s descriptive phenomenological approach was used to analyze nurses’ experiences. The consolidated criteria for reporting a qualitative research checklist were followed in this study.

**Results:**

The data analysis led to the extraction of the 5 themes and 19 subthemes. The themes identified for the study were competence, the emotional universe, stress resources, the meaning of nursing care, and profoundly affecting events. The study results show that the nurses expressed that having gained much experience in intensive care units and working there has contributed significantly to their professional development.

**Conclusion:**

It was indicated that the nurses had meaningful, caring experiences in intensive care units, which were perceived, however, as stressful experiences as well. The study has important implications for nurses, faculty members, and administrators to gain positive care experiences in terms of intensive care units.

## RESUMEN

**Objetivo:**

Describir el significado atribuido a la experiencia clínica de los enfermeros en una unidad de cuidados intensivos médico-quirúrgicos en la República Turca del Norte de Chipre.

**Método:**

El estudio cualitativo se llevó a cabo en dos unidades de cuidados intensivos médico-quirúrgicos de un hospital universitario. Los datos fueron recolectados a través de entrevistas en profundidad con 17 enfermeros. Se utilizó el enfoque fenomenológico descriptivo de Giorgi para analizar las experiencias de los enfermeros. En este estudio se siguieron los criterios consolidados para reportar la lista de verificación de investigación cualitativa.

**Resultado:**

El análisis de datos dio como resultado 5 temas y 19 subtemas. Los temas identificados para el estudio fueron competencia, universo emocional, recursos de estrés, significado del cuidado de enfermería y eventos con efectos profundos. Al final, los enfermeros expresaron que el estudio les permitió ganar más experiencia en las unidades de cuidados intensivos y contribuyó mucho para su desarrollo profesional.

**Conclusión:**

Se observó que los enfermeros tuvieron experiencias significativas de cuidado en unidades de cuidados intensivos, pero evaluaron estas experiencias como muy estresantes. El estudio tiene implicaciones importantes para que los enfermeros, los docentes y los administradores adquieran experiencias de atención positivas en las unidades de cuidados intensivos.

## RESUMO

**Objetivo:**

Descrever o significado atribuído à experiência clínica de enfermeiros em unidades de terapia intensiva médico-cirúrgica no norte de Chipre.

**Método:**

O estudo qualitativo foi realizado em duas unidades de terapia intensiva em um hospital universitário. Os dados foram coletados através de entrevistas em profundidade com 17 enfermeiros. Para analisar as experiências dos enfermeiros, foi utilizada a abordagem descritiva fenomenológica de Giorgi. Neste estudo, foram seguidos os critérios consolidados para relatar a lista de verificação da pesquisa qualitativa.

**Resultados:**

A análise de dados levou à extração de cinco temas e 19 subtemas. Os temas identificados para o estudo foram competência, universo emocional, recursos de estresse, significado dos cuidados de enfermagem e eventos profundamente afetados. Os resultados foram que enfermeiros expressaram adquirir muita experiência nas unidades de terapia intensiva e que seu trabalho nelas contribuiu muito para seu desenvolvimento profissional.

**Conclusão:**

Foi indicado que os enfermeiros tiveram experiências significativas de cuidado nas unidades de terapia intensiva, mas que, no entanto, também foram percebidas como experiências estressantes. O estudo tem implicações importantes para enfermeiros, membros de corpos docentes e administradores, que podem obter experiências positivas em termos de cuidados em unidades de terapia intensiva.

## INTRODUCTION

Intensive care units (ICU) provide life-sustaining care to critically ill patients (CIP)^(1,2)^. Critical conditions, clinical instability, the need for monitoring, ethical dilemmas in patient care, a tension-charged atmosphere, a high number of interventions, and the probability of occurrence of adverse events, mortality, and morbidity make the patients’ care complex and challenging in ICUs^(3,4)^. Such patients receive aggressive care and are monitored throughout their ICU treatment^(3,5)^. To meet the specific needs of CIPs, ICUs nurses provide highly specialized care using advanced technology^(3)^. Besides, critical illness, increased workload, and the survival of patients are other factors that may influence ICU nurses’ actions and emotions^(4,5)^.

In addition to providing care for CIPs, working with unsafe staff levels, insufficient resources, participation in end-of-life discussions, prolonging life with artificial assist devices, and the risk of providing improper care can be stressful for ICU nurses^(6)^. In these stressful conditions, nurses may feel powerless and prone to moral distress^(7)^.

In ICUs, the quality of nursing care depends on the qualification of professionals^(8)^. Nurses care for patients in an ICU environment where they accumulate experience in highly dynamic processes^(3,4)^. Develope an understanding of the subjective perceptions of nurses serving in such units is essential to improving the quality of nursing care^(5)^. Studies investigating nurses’ nursing care experiences in ICU settings describe how caring originates in nurses’ feelings of empathy, anxiety, frustration, fear, insecurity, impotence sensitivity, and concern for the patients^(7,9)^. A limited number of researchers have investigated an in-depth understanding of the CIPs clinical experiences of ICUs nurses^(4,10)^.

The objective of this study was to describe the meaning attributed to the clinical experience of nurses in a medical-surgical ICU in Northern Cyprus.

## METHOD

### Design of Study

This study was performed with a qualitative design. A descriptive phenomenological approach was used to obtain deeper insights into the nursing staff’s experiences in medical-surgical ICUs^(11,12)^. In phenomenology, sampling is the selection of individuals from a population to be researched; the individuals to participate in research should have experience or be involved in the phenomenon to be investigated^(13)^. Phenomenology allows making an in-depth examination of the universal essences of a phenomenon. It is believed that a person’s experiences are related to the person’s perception and give meaning to a particular event^(6)^. Consolidated criteria for reporting qualitative research (COREQ) checklist have been adhered to in this study^(14)^.

### Local

This study was conducted in two medical-surgical ICUs in a university hospital in Northern Cyprus. The university hospital involved in the project has a total of 137 beds, with 108 clinical beds and 29 ICU beds. Nurses care for severe intoxication, multiple trauma, severe neurological damage, stroke, acute brain perfusion disorders, gastrointestinal system bleeding, liver insufficiency, lung diseases and any type of respiratory failure, serious infections, sepsis, and postoperative patients in the ICUs. On normal days, the nurse-to-patient ratio is 1:2 in the ICU. However, some days it can be 1:3 in the summer season, particularly with a high workload.

### Selection Criteria and Population

The inclusion criteria were that participants should be employed in medical-surgical ICUs for at least one year and speak the Turkish Language. The exclusion criteria were caring CIPs in the ICUs for less than one year. The electronic mail addresses of the participants who met the inclusion criteria were obtained from the hospital administration. Afterward, e-mails were sent with detailed information about the research and voluntary participation. The interview schedule was agreed on if the participant replied to the electronic mail to participate. The first author constructed the schedule. Of a total of 33 nurses who received the electronic mail, 21 nurses chose to participate; 12 of the nurses did not reply to the electronic mail. Four participants dropped out, and interviews could not have been completed because of the urgent situation of the CIPs’ care. A convenience sample of 17 registered nurses was recruited from the medical-surgical ICUs.

### Data Collection

The research data were gathered using an information form containing demographic data and a semi-structured interview questionnaire^(2,6,10)^, both created by the researchers and evaluated by five experts in the nursing field. After the pilot interview, no questions were excluded from the study (Table 1).

**Table 1.** A semi-structured and in-depth interview guide – Nicosia, Cyprus, 2016.

Questions
1. What are your experiences with working in an intensive care unit?
2. What do you feel when you give care to a patient having intensive care?
3. Do you remember any special event that affected you deeply while working in this unit?
4. What did you feel about the event or events? Could you please give more details?
5. What is the meaning of patient care for a nurse working in an intensive care unit?

The study was conducted using individual face-to-face and in-depth interviews. The interviews were carried out by the first author, who had an ICU nursing background and had received training in qualitative interview skills. The data collection was performed in a private room in the ICU. During some shifts, interviews had to be interrupted due to CIPs’ conditions and urgent situations.

Data collection was conducted for five months, from April to August 2017, using an audio recorder and making field notes with the consent of the participants. The authors did not have a relationship with any of them. Seventeen interviews were performed, ranging from 18 to 23 minutes. After the interviews were completed, data saturation was discussed by three authors.

### Data Analysis and Treatment

Demographic data were analyzed using the Statistical Package for the Social Sciences (SPSS Version 25). The analysis included mean, minimum, maximum values, and percentages.

The audio recordings were listened to several times, typed word-by-word in Microsoft Office 365 Word Document, and added field notes within 24 h by the first author. The audio recordings were transcribed and translated from Turkish into English by a research associate who could speak Turkish and English and had experience in qualitative studies and checked by all authors. It was important to translate the transcribed verbatim into English because all the participants of this study spoke and understood Turkish. Transcribed verbatim was returned to participants for comment and correction. Participants added no comments or corrections.

In order to analyze the content transcribed verbatim was managed by MAXQDA (Version 10). Giorgi’s four-stage approach was used to analyze the experiences of ICU nurses thematically. Stages were reading full descriptions, determining descriptions into meaning units, transforming meaning units, and determining and integrating features into structures of phenomena^(11,12)^. The findings are presented as themes and subthemes using a phenomenological approach. Three authors undertook independent analysis and met to agree on themes and subthemes. In this way, interpretive robustness was enhanced. The authors were female lecturers, had doctorates, had nursing backgrounds, and had received education in qualitative research during their master’s and doctoral educations.

### Ethical Aspects

The study was conducted according to the Principles of the Declaration of Helsinki (Ethical Principles for Medical Research Involving Human Subjects). Before conducting the study, ethical clearance was obtained from the university’s ethics review board and the university hospital’s permission (Reference number: YDU/2016/41-338). In addition, all the nurses participating in the study on a volunteer basis were informed about the research by explaining the study purpose, and their verbal and written permission was obtained before starting the data collection.

The validity and reliability of the present study were achieved when the authors rigorously followed many strategies in the study process. The concept of trustworthiness contributed to and built the reliability and validity of the study, thus ensuring rigor. In this qualitative study, four criteria: credibility, dependability, conformability, and transferability, were used to evaluate trustworthiness. Credibility was performed by interviewing participants in a reflective dialogue approach, data analysis, and member checking of participants following data analysis. Dependability was achieved by establishing audit trails, involving data analysis, and making decisions by three researchers. Confirmability was ensured by audio recording and detailed documentation of the study process, and transferability was maintained by in-depth descriptions describing the participants’ caring experiences^(15,16)^.

Regarding the suitability of ethical principles, in the results part of the study, the participants were expressed with participant numbers at the end of the sentences in which they indicated their testimonials (P1, P2, etc.).

## RESULTS

Most participants were female, and the average age was 27.6 ± 4.7 years. The mean work experience of the participants was 4.6 ± 2.8 years. Fifteen participants (88.4%) had a degree of Bachelor of Science in Nursing (Table 2).

**Table 2.** Demographic data (N = 17) – Nicosia, Cyprus, 2017.

Variables	N(%)
**Gender** Women Men	14(82.3) 3(17.6)
**Age** Mean ± SD (range) years	27.6 ± 4.7 (23–40) years
**Marital status** Single Married	11(64.4) 6(35.6)
**Having children** No Yes	14(82.4) 3(17.6)
**Educational background** Bachelor of Science Diploma in Nursing College	15(88.4) 1(5.8) 1(5.8)
**Nursing specialty** Coronary ICU General ICU	10(58.8) 7(42.2)
**Years of experience in nursing** Mean ± SD (range) years	4.6 ± 2.8 (1–19) years
**Years of experience in ICU** Mean ± SD (range) years	2.l ± 2.8 (1–7) years
**Working status** Day shift Day shift and night shift Night shift	2(11.7) 7(41.2) 8(47.1)

### Themes and Subthemes

After rigorous data analysis, five themes and nineteen subthemes emerged, which are illustrated in Table 3.

**Table 3.** Themes and subthemes – Nicosia, Cyprus, 2017.

Themes	Subthemes
**Competence**	Holistic care Nursing Practice Learning
**Emotional universe**	Empathy Happiness Sadness Fear Patience
**Stress resources**	Critically ill patients Family members Responsibilities Difficulties
**Meaning of nursing care**	Uniqueness Monitoring, early intervention, and nursing care Communication
**Deeply affecting events**	Experiencing the death of a patient Patient safety

### Competence

This theme consists of three subthemes: Holistic care, nursing practice, and learning. Most of the nurses expressed having positive experiences regarding this theme.

***Holistic care:*** Most nurses agreed that providing holistic care was an essential part of nursing care.

*While providing care for CIPs in an ICU, we not only measure the blood pressure and pulse rate of the patients, but we also monitor and interpret all the progress (P1).*

***Nursing practice:*** The nurses expressed having gained much experience in the ICU:

*Here in the ICU, I have gained much experience. Now I can say there is no aspect of nursing care I have not seen and practiced (P2).*

*Most patients treated in the ICU are unconscious; they must be provided with continuous care (P3).*

***Learning:*** The nurses reported that working in an ICU has contributed significantly to their professional development.

*In the ICU, you can experience a greater number of cases, all more different from the ones in the clinical environment, which have significantly contributed to my knowledge (P4).*

*Here I have learned how to handle a patient in an integrated manner; I have gained more skill and knowledge (P5).*

### Emotional Universe

This theme consisted of the subthemes of empathy, happiness, sadness, fear, and patience. Most of the nurses in this study indicated positive emotional reactions to patient care in the ICU.

***Empathy:*** Most nurses reported difficulty approaching patients with empathy in the initial stages of their professional life but overcame this feeling over time. They also said that sympathy could sometimes replace an emphatic approach.

*One tries to restrain the impulse to approach the patients with sympathy. In the initial stages of my work in the ICU, I felt that I began to distance myself from an empathic approach. However, after a long time of service in the unit, I began to adopt an empathic approach toward patients (P1).*

*In fact, it might seem unethical, but I usually look at the patients as my relatives. In other words, I plan the nursing care, always considering how I would treat them if they were my relatives or how the patients would like to be cared for, and I plan every detail (P6).*

***Happiness:*** The nurses expressed being happy to care for patients, reporting that they feel good, especially when their patients are discharged from the hospital.

*Helping patients and giving them hope makes us feel good. A grateful thank you or a smile of a patient or experiencing the recovery of patients makes us feel good and happy (P7).*

*Caring for critically ill patients gives exceptional satisfaction and brings greater happiness when we see them recovering and then being discharged home (P8).*

***Sadness:*** The nurses expressed a sense of sadness due to the critical situation of the patients. They reported that they also felt unhappy when they could not give sufficient care to the patients.

*In general, we take great care to do everything in compliance with the related treatment schedules. However, sometimes we cannot do it due to an excessive number of patients, and we feel unhappiness when we cannot do it for any reason whatsoever (P3).*

*We feel a sense of great sadness when patients die; there were cases where we burst into tears when a patient died after a long stay in the unit (P8).*

***Fear:*** Some nurses reported that they felt fear due to the critical condition of patients.

*During the initial phase of my work here, I felt a sense of fear because most of the patients were in an unconscious condition, and they needed to be put on a ventilator to support breathing. The machines and equipment frightened me (P3).*

*The ICU is not like the clinic. I was terrified when I experienced the first cardiac arrest case; I got very uneasy because I didn’t know what to do (P5).*

***Patience:*** A nurse said that patience toward CIP is an aspect challenging to tackle.

*Giving care to some patients can strain us; however, as nursing staff members, we must be patient despite everything. If we lose our patience, the care we provide will be of poor quality, and it could also present a risk to the privacy of patients (P4).*

### Stress Resources

This theme consists of four subthemes: CIPs, family members, responsibilities, and difficulties. Most of the nurses stated that providing support to CIPs and their family members was an essential part of nursing care but that it could also be a resource of stress from time to time.

***Critically ill patients:*** The nurses expressed that the unstable general condition of the CIPs cared for in the ICU is the most significant source of stress for them.

*We have too great a burden on our shoulders because we are caring here for CIPs; here, everything can happen at any moment… any patient receiving intensive care can suddenly have a cardiac arrest or die (P9)*.

***Family members:*** The nurses reported that the reactions of family members of CIPs were a source of stress.

*Sometimes they complain about processes that usually progress, depending on their perceptions. They sometimes consider that the patient is not sufficiently cared… it is an offending situation for us (P8).*

***Responsibilities:*** The nurses reported that a sense of responsibility was another source of strain for them.

*Here the conditions are very different. We must have a strong awareness and perception of what is happening in the unit. The fact that even a minor failure can cost the life of a patient persistently haunted my mind. I said to myself: Leave all your private life at the unit’s door, and you can live on after the shift. Here you must consider even the most minor detail (P1).*

***Difficulties:*** The nurses expressed that the difficulties that arose during the care could also be a source of stress.

*When there are too many patients, it becomes difficult to care for all the patients as required, especially for the bedridden patients and those with decubitus (P7).*

### Meaning of Nursing Care

The theme meaning of nursing care consists of uniqueness, monitoring, early intervention, nursing care, and communication. Most of the nurses in this study underlined the unique character of patient care in an ICU.

***Uniqueness:*** The nurses underlined the unique nature of the patient care in an ICU arising from the critical condition of patients and said that the care provided in these units had to be unique and patient-specific.

*Caring for patients in an ICU is a divine job for me, so the better the care we give them, the better their chances to hold on to life (P4).*

*I think the care I provide to the patients here is the true metier of nursing care in the real sense (P6).*

*What we experience here in the ICU are experiences of a specific character, all experiences gained with particular patients. It was an incident that made me the sense that there is a thin line between life and death (P10).*

***Monitoring, early intervention, and nursing care:*** The nurses reported that close monitoring, early intervention and treatment, and nursing care are aspects of essential importance in an ICU.

*The patients treated in the ICU need to be monitored more closely than those in other departments. More rapid intervention and effective treatment are provided to the patients treated in an ICU (P1).*

*Defining the priorities of each patient is of great importance in an ICU. In other words, providing treatment only does not mean effective intensive care nursing. Only monitoring does not serve the purpose; the monitors are always available, but it is more important to recognize them, such as the aspiration need of a patient or the need for better positioning or to maintain the best hygienic conditions for patients (P6).*

***Communication:*** The nurses underlined the importance of communication in an ICU.

*It is not only about observing patients’ vital signs, treating them, and going home at the end of the shift. What is most important is to communicate with patients (P12).*

### Deeply Affecting Events

Asked about the circumstances that have profoundly affected them during their work in the ICU, the nurses said that they were deeply affected by the exitus of patients, and one nurse said it was a misuse of hand antiseptic.

***Experiencing the death of a patient:*** The nurses expressed being deeply affected by dying patients.

*I remember a patient. One night, I saw that he was covered; he had recently passed away. I felt terrible; tears filled my eyes. My hands were shaking as I took his electrocardiogram (ECG). A sense of deep sorrow hit me; it was as if a part of me had also died with him. I have never forgotten that patient; I still remember him by name because it was the first lethal exitus case I experienced (P9).*

***Patient safety:*** One nurse said that what affected her most deeply was an incident of patient safety arising from the misuse of the hand antiseptic used in the unit.

*A patient who had undergone a bypass operation had pain. The patient was also an alcoholic, which he had not reported to us. He had to take painkillers every four hours, and for this purpose, he always had a glass of water at the bedside. When I checked him, I saw that the water in the glass was cloudy. The patient had drunk the water after mixing it with the hand antiseptic on the bedside table. I felt awful when I learned it. I had a shock (P15).*

## DISCUSSION

In the present study, the caring experiences of nurses serving in the medical-surgical ICUs at a university hospital in Northern Cyprus were investigated based on the themes of competence, the emotional universe, stress resources, the meaning of nursing care, and profoundly affecting events. In the literature, there have been studies that have addressed similar themes^(1,3,4,6,17)^. It was found that the experience’s overall meaning was associated with becoming competent in an ICU^(17)^.

### Competence

The studies on the competence of ICU nurses found that presence was strongly underpinned by holistic care^(18,19)^. The lived experience of nurses caring for CIPs, with inadequate competence and support, can affect holistic care^(1)^. In this study, most nurses emphasized the importance of holistic care.

It was stated that the preference for intuition in nursing was due to a general preference for intuition and that use of nursing intuition increased with competence^(20)^. Repeated exposure to various clinical situations develops the nurses’ intuition^(17,20)^. It was also found that frequently caring for CIPs with the same or similar care needs greatly affected the nurses’ competence to notice changes in a CIP’s condition early^(21)^. Similarly, the present study indicated that the nurses’ numerous caring practices and competence significantly contributed to their professional development.

### Emotional Universe

ICU nurses provide care for the individual needs of CIPs and their family members through the integration of processes with affective and cognitive skills and the aspect of action^(21)^. They are physically, cognitively, and emotionally demanding while meeting the needs of CIPs^(8,22)^. Primarily they may express negative emotions such as anger, sadness, frustration, and guilt when describing their feelings. ICU nurses who experience such emotions may change positions and leave the profession entirely^(23)^. Some studies indicated that ICUs generate a complex emotional universe with opposing affective experiences for nurses^(7,23)^. Moreover, most newly graduated and young nurses may experience an extensive range of emotions^(24)^.

It was found that the nurses perceive empathy as a way of being with the patient^(8,9,18)^. They may obtain satisfaction and well-being from an empathic connection with the CIPs^(7)^. This was described as an effort to imagine and try to understand what the individual CIPs are experiencing and feeling and determine what their caring needs are^(22)^. It was indicated that fear, insecurity, anxiety, frustration, and impotence were highlighted emotions and very close to the anguish and stress experienced by ICUs nurses in the honeymoon phase particularly^(7,17,21)^. In the present study, the emotional universe expressed by the nurses about patient care in the ICUs was empathy, happiness, sadness, fear, and patience. The study’s results match the other studies that nurses had a complex emotional universe^(7,22–25)^. This emotional complexity may cause job stress and burnout for ICU nurses participating in our study.

### Stress Resources

In the present study, most participants expressed that providing support to CIPs and their family members was essential to providing nursing care but that they sometimes could be a source of stress.

In the studies, it was stated that the family members at all times place the CIPs in first place in their life and always want to be close to their patients, with or without participating in the care^(7,26)^. Nurses reported that sometimes families have unrealistically high expectations of caregiving of nurses^(6)^. It was indicated that CIPs’ family members are almost as central in their care delivery as the patients themselves^(7)^. The contribution of the family members to the CIPs care process is thought to be a source of stress for the nurse while supporting the patient. Furthermore, participants indicated the importance of responding to CIPs’ family members, the need for information to reduce their stress, and the necessity to explain what is happening^(19,27)^.

The nurses reported that stress sources were the sense of responsibility for the condition of patients, an excess number of patients, and family members of patients. Similarly, studies reported that aspects such as staff shortages, lack of time, inability to communicate, and stress in caring for CIPs were described by the participants in their study as factors of missed care^(26,27)^. A 1:1 or 1:2 nurse-to-patient ratios are standard for all ventilated and non-ventilated patients in the ICUs. The fact that the nurse-patient ratio is above these rates could be defined as the high workload of the nurse staff. This is a reason for stress and missed care^(8)^.

### Meaning of Nursing Care

The nurses said that aspects such as uniqueness of care, monitoring, early intervention, and nursing care and communication constituted the meaning of nursing care for ICU nurses. The nurses underlined the unique nature of CIP care in an ICU arising from the critical condition of patients. Studies indicated that caring for the CIP was equated to keeping the patient alive in ICU and that once this was achieved, the nurse could then concentrate on the other aspects of caring^(1,2,10)^.

The nurses participating in the study expressed that they considered the interpersonal aspects of caring important. When a nurse identifies herself or himself with the patient as an individual unique person, it creates emotional involvement, leading to a sense of ‘being close’ to the patient^(10)^. However, it was indicated that being close to patients leads to feelings of love, awe, and compassion^(28)^. Our findings are consistent with the study on the importance of providing emotional support when communicating with family members^(27)^. In a study on the communication skills of oncology nurses, it was expressed that good communication and psychological nursing care made it possible to create an ambiance marked with comfort as a significant aspect of empathetic caring^(25)^.

### Deeply Affecting Events

In the present study, asked about the circumstances that deeply affected them during their work in the ICU, the nurses said that the event that most affected them was the death of a patient. It was determined that young nurses were affected more than experienced nurses^(7,17,24)^. Nurses are not emotionally well prepared to deal with the patient’s imminent death^(29)^. In these situations, nurses distance themselves emotionally. Because the undergraduate nursing courses mainly focus on saving lives, caring for, preventing, and promoting health^(30)^. Similar to the present study, nurses expressed in other studies that they often shed tears with the family while providing terminal care^(6)^.

The complexity of CIPs’ nursing care processes in the ICU makes the system vulnerable and prone to error, creating an environment that may profoundly affect the caring nursing staff^(3,4,30)^. Similarly, one nurse participating in our study said that what affected her most deeply was an incident involving patient safety arising from the misuse of the hand antiseptic used in the unit. On the other hand, it was indicated that dealing with critical situations was essential to nursing care^(22)^.

This study has some limitations. The limitations linked with qualitative research apply to this study. The study population is quite selective, as participants have been recruited from a university hospital. For this reason, the results are not generalizable. Moreover, a further limitation may occur in the form of data analysis. The thematic analysis is based on the articulated phrases; therefore, unexpressed attitudes and non-verbal information have not been included.

## CONCLUSION

In the present study, the caring experiences of nurses serving in ICUs at the university hospital in Northern Cyprus were investigated using the study themes of competence, the emotional universe, stress resources, the meaning of nursing care, and deeply affecting events. The present study’s findings showed that the nurses had positive and meaningful caring experiences with CIPs in the ICUs. This has contributed significantly to the nurses’ professional and individual development but is also perceived as a stressful experience.
